# Activated Amorphous Carbon With High-Porosity Derived From Camellia Pollen Grains as Anode Materials for Lithium/Sodium Ion Batteries

**DOI:** 10.3389/fchem.2018.00366

**Published:** 2018-09-04

**Authors:** Kaiqi Xu, Yunsha Li, Jiawen Xiong, Xing Ou, Wei Su, Guobin Zhong, Chenghao Yang

**Affiliations:** ^1^Electric Power Research Institute of Guangdong Power Grid Co., Ltd., Guangzhou, China; ^2^Guangzhou Key Laboratory for Surface Chemistry of Energy Materials, New Energy Research Institute, School of Environment and Energy, South China University of Technology, Guangzhou, China

**Keywords:** activated amorphous carbon, anode, high porosity, lithium ion batteries, sodium ion batteries

## Abstract

Carbonaceous anode materials are commonly utilized in the energy storage systems, while their unsatisfied electrochemical performances hardly meet the increasing requirements for advanced anode materials. Here, activated amorphous carbon (AAC) is synthesized by carbonizing renewable camellia pollen grains with naturally hierarchical structure, which not only maintains abundant micro- and mesopores with surprising specific surface area (660 m^2^ g^−1^), but also enlarges the interlayer spacing from 0.352 to 0.4 nm, effectively facilitating ions transport, intercalation, and adsorption. Benefiting from such unique characteristic, AAC exhibits 691.7 mAh g^−1^ after 1200 cycles at 2 A g^−1^, and achieves 459.7, 335.4, 288.7, 251.7, and 213.5 mAh g^−1^ at 0.1, 0.5, 1, 2, 5 A g^−1^ in rate response for lithium-ion batteries (LIBs). Additionally, reversible capacities of 324.8, 321.6, 312.1, 298.9, 282.3, 272.4 mAh g^−1^ at various rates of 0.1, 0.2, 0.5, 1, 2, 5 A g^−1^ are preserved for sodium-ion batteries (SIBs). The results reveal that the AAC anode derived from camellia pollen grains can display excellent cyclic life and superior rate performances, endowing the infinite potential to extend its applications in LIBs and SIBs.

## Introduction

The emergence and rapid development of portable electronic devices, electric vehicles and renewable energy industries urge preeminent large-scale energy storage and conversion systems, which exhibit superior energy density and specific capacity as well as phenomenal life-span with trustworthy safety (Hannan et al., 2017; Zhang et al., 2017). Among prevalent energy storage systems, lithium ion batteries (LIBs) and sodium ion batteries (SIBs) attract most attentions owing to their high working potential and extraordinary storage capacity (Shen et al., 2018). It is well-known that in order to promote practical applications of LIBs and SIBs, developing high-performing electrode materials are inevitable (Zheng et al., 2015; Luo et al., 2016). With increasing improvements in cathode materials, commercial graphite as the mainstream anode material gradually becomes dissatisfactory due to its limited theoretical capacity (372 mAh g^−1^ for LIBs) (Hou et al., 2015; Zhang et al., 2016b). Besides, the highly regular distance between graphene layer (0.335 nm) can hardly accommodate the size of Na^+^, prohibiting the possibility to form stable Na-intercalation compounds (Irisarri et al., 2015; Yang et al., 2017). Though graphite hinders the improvement of LIBs and SIBs, carbonaceous materials still consumingly appeal to researchers, thanks to their significant advantages of low lithium/sodium ion insertion/desertion potential, highly structural stability, cost-efficiency, and sufficient resources (Hou et al., 2017a,b).

Amorphous carbon, compared to graphite, can reach higher specific capacity with enlarged interlayer distance and shortened ion transportation paths. Owing to environment amiability, sustainability and cost efficiency, ta large amount of diverse selectable biomass materials, such as protein (Li et al., 2013), peanut shell (Lv et al., 2015), bee pollen grain (Tang et al., 2016), rice husk (Zhang et al., 2016a), cotton (Li et al., 2016; Xiong et al., 2018; Yang et al., 2018), garlic skin (Zhang et al., 2018c), have been investigated in depth of their electrochemical capabilities. Generally, biomass-derived amorphous carbon can be synthesized by directly pyrolysis (Li et al., 2016)^−^(Wang et al., 2015) or hydrothermal treatment (Li et al., 2015). It is noted that most of the biomass-derived carbon can successful maintain the uniform sphere shape, inherent textile and hollow tubes. Despite of advantages of biomass-derived carbon, it is convinced that its electrochemical potential has not been ultimately exploited. To address this challenge, researchers have exerted their efforts on constructing inner microstructures and modifying surface properties of biomass-derived carbon by utilizing the activation methods (Gu et al., 2014; Wu et al., 2016a). Noticeably, chemical activation by mixing carbon materials and chemical reagents, KOH for instance, is less energy-consuming than physical activation resulting from the relatively low anneal temperature (Gao et al., 2016).

Pollen grains, distinctively, presenting uniformly discrete particle distribution and divergent particle shapes from various kinds of flowers, are usually employed in synthesis of various materials as templates (Chen et al., 2016). When regarded as anode for LIBs, cattail pollen carbon after physically activating exhibits 382 mAh g^−1^ at 25°C at rate of 37.2 mA g^−1^, distinguishing itself as a competitive candidate in anode materials beyond graphite (Tang et al., 2016). Impractical test current density is reason of the slightly better capacity, which still need further improvements. Therefore, further exploration of beneficiated activations for fabricating pollen grain's microstructure is required.

In this work, taking the advantage of pollen grains, we put forward a homogeneous and tunable synthetic method for activated amorphous carbon (AAC) derived from camellia pollen grains. Pollen grains were activated through chemical activation using KOH as activating reagent (Su et al., 2017), followed by a two-stage carbonization process. The eventual product is identified as poly-porous amorphous carbon with enhanced specific surface area around 660 m^2^ g^−1^ and enlarged layer distance from 0.35 to 0.4 nm, which contributes to advanced specific capacities and cyclic performances with excellent rate when acting as anode materials for LIBs and SIBs. The outstanding characteristics provide camellia-pollen-grain-derived carbon with the opportunity to meet the increasing demand of advanced storage devices.

## Experimental

### Material preparation

The activated amorphous carbon was synthesized by annealing camellia pollen grain. After rinsing with acetone and deionized water, 3 g raw pollen grains were mixed with 80 mL KOH solution (0.025 g mL^−1^) and stirred for 6 h at 40°C, following by drying process at 80°C. The obtained pollen grains were then transferred into a tube furnace and underwent the two-stage heat treatment under N_2_ atmosphere, which suggested maintaining temperature at 400°C for 1 h at the first stage and 800°C for 3 h at the second stage with the fixed heating rate of 5°C/min for the entire calcination. Finally, the sample was etched by 3M HCL and thoroughly washed with deionized water and desiccated at 80°C for 24 h. The as-prepared activated amorphous carbon was denoted as AAC.

For comparison, another 3 g raw pollen grains were directly annealed through two-stage heat treatment aforementioned to acquire pristine amorphous carbon, denoting as AC.

### Material characterizations

Scanning electron microscope (SEM, FEIQuanta 200 FEG) and transmission electron microscope (TEM, Tecnai G2 F20 S-TWIN, Japan) were performed to unveil the morphology and microscopic structure of both samples. To gain the intrinsic features of the samples, X-ray diffraction (XRD, Bruker D8 Advance, Germany) (Cu, Kα, λ = 1.5405 Å), Raman spectrometer (JOBIN-Yvon HR800) and X-ray photoelectron spectroscopy (XPS, Thermo/ESCALAB 250XI) were conducted. N_2_ adsorption/desorption isotherms for calculation of specific surface area (SSA) and accumulative pore volume based on Brunauer-Emmett-Teller and density functional theory methods was carried out on ASAP 2020 Micromeritics.

### Electrochemical measurements

The working electrode was fabricated using copper foil covered with slurry comprised of 70 wt % of AAC or AC, 20 wt % of PVDF binder and 10 wt % carbon black. Subsequently the electrode is dried out with 0.5 mg cm^−2^ average active material loading to assemble CR2032 coin cells together with PE/PP films or glass-fiber papers as separators, metal lithium or sodium foils as counter electrodes and corresponding electrolyte (1 M LiPF_6_ or 1 M NaCF_3_SO_3_ in ethylene carbonate, dimethyl carbonate and ethyl methyl carbonate = 1:1:1(v: v: v) for lithium ion batteries and sodium ion batteries, respectfully). CV and EIS results were attributed to the employment of CHI660A electrochemical workstations while galvanostatic charge/discharge profiles were resulted from the measurements of LAND CT2001A battery-testing instruments.

## Results and discussions

**Figure 1:**
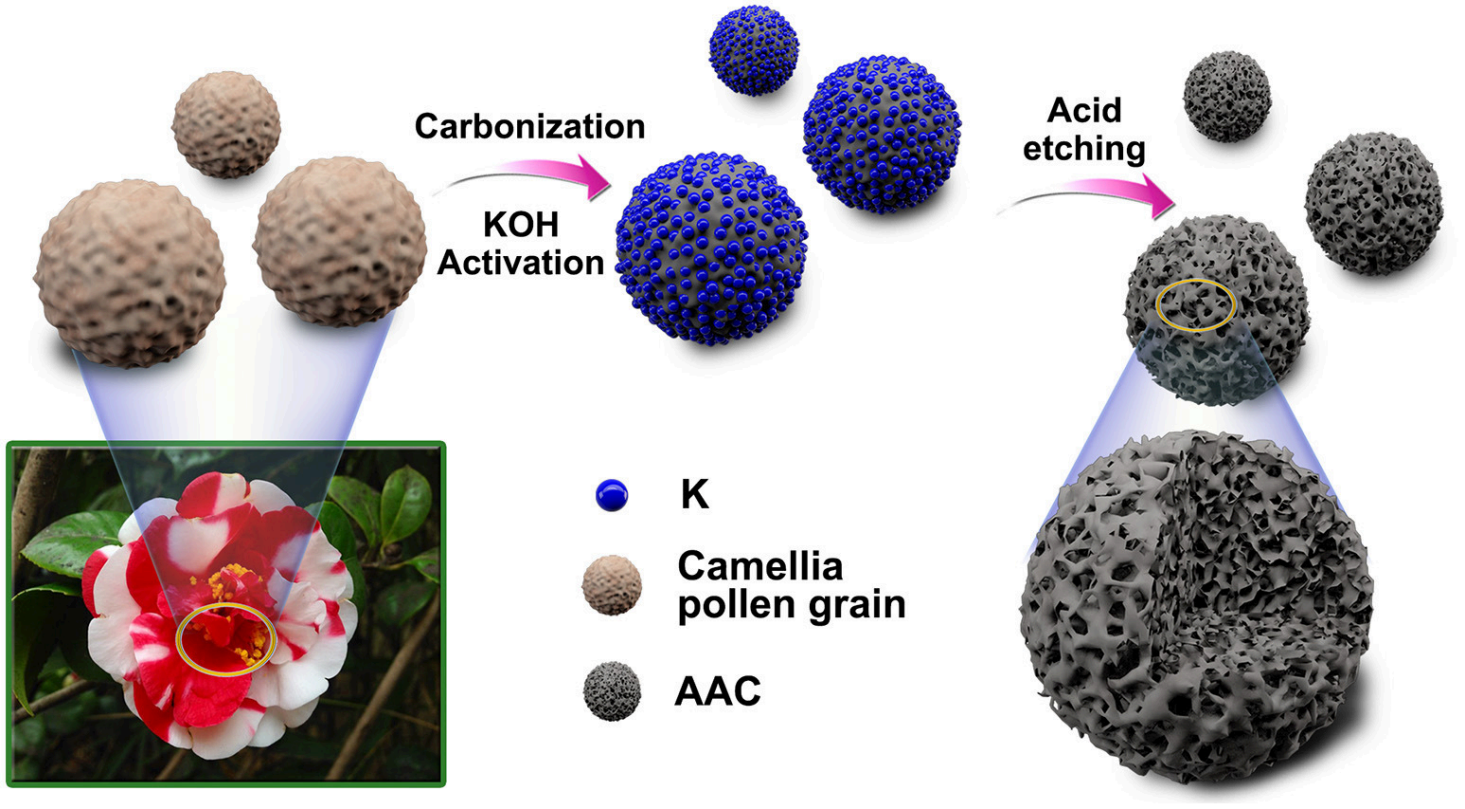
Schematic illustration of the synthesis process for the activated amorphous carbon derived from camellia pollen grains.

**Figure 2:**
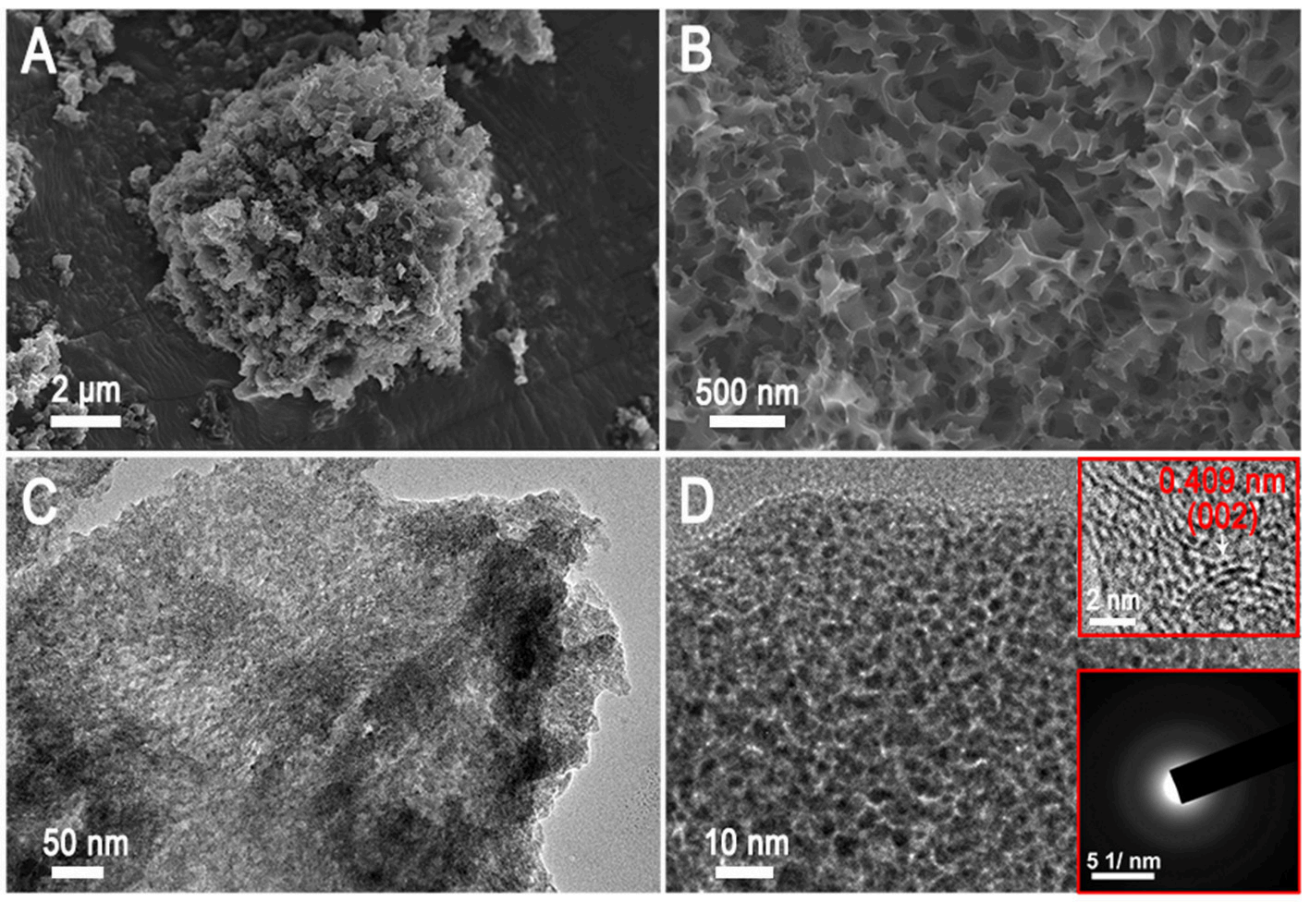
**(A, B)** SEM images and **(C, D)** TEM images of AAC (the inner figures are SAED and HRTEM).

The overall synthesis of AAC is shown in Figure 1. The KOH activation followed by carbonization and acid etching process are involved in this strategy to introduce poly-pores into the interior structures of camellia pollen grains (details in experimentally section). The microscopic morphologies of the original pollen grains, AC and AAC are analyzed *via* SEM, TEM and HRTEM images (Figure 2 and Figure S1). Camellia pollen grains are consisted of irregular spheres with rough surfaces, and the uniform diameter is about 30 μm (Figures S1A,B). In comparison with AC (Figures S1C,D), Figures 2A,B visualizes that, in morphology, AAC possesses smooth surface, vast amount of mesopores and micropores, while primitive bulk shape is well-maintained, proving the effective function of KOH agent as impetus aiming at high porosity. Consistency can be found in corresponding TEM images (Figure 2C), in which explicit micro- and mesopores morphology. Moreover, it is noticeable in HRTEM (the inset figure of Figure 2D) that AAC is comprised of turbostratic nanodomains with ~0.4 nm interlayer distance in average graphene interlayers. The evidence of dispersed diffraction ring in selected area electron diffraction pattern (SAED) can be observed, consistent with the HRTEM results, confirming the amorphous characteristic of AAC.

**Figure 3:**
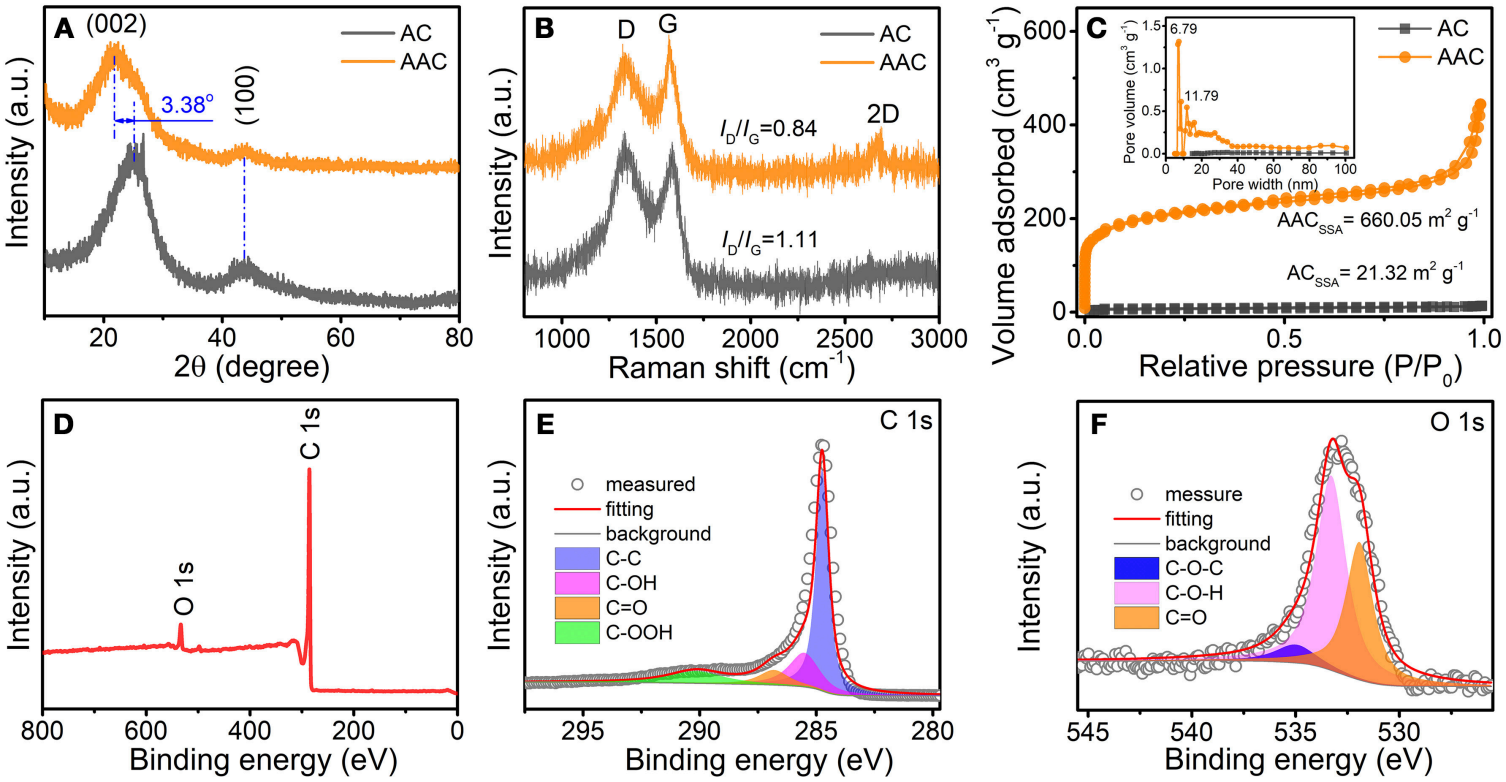
**(A)** XRD patterns, **(B)** Raman spectra, and **(C)** N_2_ sorption isotherms (inner figure is pore size distributions) of AAC and AC; **(D)** XPS survey scan spectrum of AAC; **(E,F)** high resolution XPS spectra of **(E)** C 1s and **(F)** O 1s.

To gain a deeper insight of the composition and crystal structure of both samples, XRD patterns, and Raman spectra are investigated as displayed in Figures 3A–C. The broad peaks in XRD pattern referring to (002) facet are detected at 2θ of 21.8° and 25° for AAC and AC, respectively (Figure 2A), confirming the amorphous property of both samples. It is noted that the left shift of 2θ for AAC is ascribed to the enlarged interlayer. According to Bragg equation (Ou et al., 2016), interlayer distance between graphene layer can be calculated to be 0.409 and 0.352 nm for AAC and AC, respectively, both larger than 0.335 nm for graphite (Hou et al., 2017a). Such appealing expansion for AAC should be ascribed to KOH activation mechanism, which is explained by three steps as followed: (i) Firstly potassium-containing compounds commence etching carbon substrate to form the initial porous framework, which is called chemical activation. (ii) Then H_2_O and CO_2_ escape to impel the increment of porosity for physical activation. (iii) Metallic K permeates into carbon matrices, achieving the larger graphene interlayer distance, resulting in the lattices expansion (Romanos et al., 2012; Wang and Kaskel, 2012). The enlarged interlayer distance has favorable influences on the electrochemical performance of AAC, which enables reversible intercalation for Li^+^/Na^+^ ion and well maintain the structural integrity at the same time (Wen et al., 2014; Kim et al., 2017; Zou et al., 2017; Yang et al., 2018; Zhu et al., 2018). Subsequently, D-band representing defects induction at ~1334 cm^−1^ and G-band representing in-plane vibration at ~1577 cm^−1^ are located in Raman spectra (Figure 3B), whereby the I_D_/I_G_ can be calculated as 0.84 for AAC and 1.11 for AC. Furthermore, based on the I_D_/I_G_ ratio, the AAC is of higher graphitization degree, providing a crucial foundation for improving electrical conductivity (Lv et al., 2015; Zhang et al., 2018c).

In addition, N_2_ adsorption/desorption isotherms measurement and differential pore volume distributions are tested for both sample in Figure 3C. The specific surface area (SSA) of AAC dramatically increases from 21.32 to 660.05 m^2^ g^−1^. While concurrently the accumulative pore volume of AAC scales up to 0.29 cm^3^ g^−1^, overwhelming larger than the AC of merely 0.01253 cm^3^ g^−1^. Unlike AC showing negligible few pores, AAC contains ample micropores and mesopores concentrating at ~6 and 11 nm. The improved SSA and porosity, resulted from synergic effect between the chemical conversion of KOH and formation of H_2_O and CO_2_, offers not only extra active sites for Li^+^/Na^+^ ion but also shortened ion transportation path with reduced inner resistance (Yu et al., 2016). To understand the existing chemical states of all elements in AAC, XPS measurement is characterized. Judging from XPS curve as displayed in Figure 3D, the peaks at 285.08 and 534.08 eV are attributed to C and O elements, yielding an occupancy of 94.56 and rest 5.44%, respectively. Individually, as displayed in Figures 3E,F, four predominant peaks are specified as C-C (284.78 eV), C-OH (285.48 eV), C=O (286.88 eV), and C-OOH (290.08 eV) in deconvoluted C 1s spectrum. Besides, three apparent peaks in deconvoluted O 1s spectrum are assigned to the representative C=O (531.88 eV), C-O-H (533.28 eV), and C-O-C (534.98 eV) (Lin et al., 2016; Li et al., 2017a).

**Figure 4:**
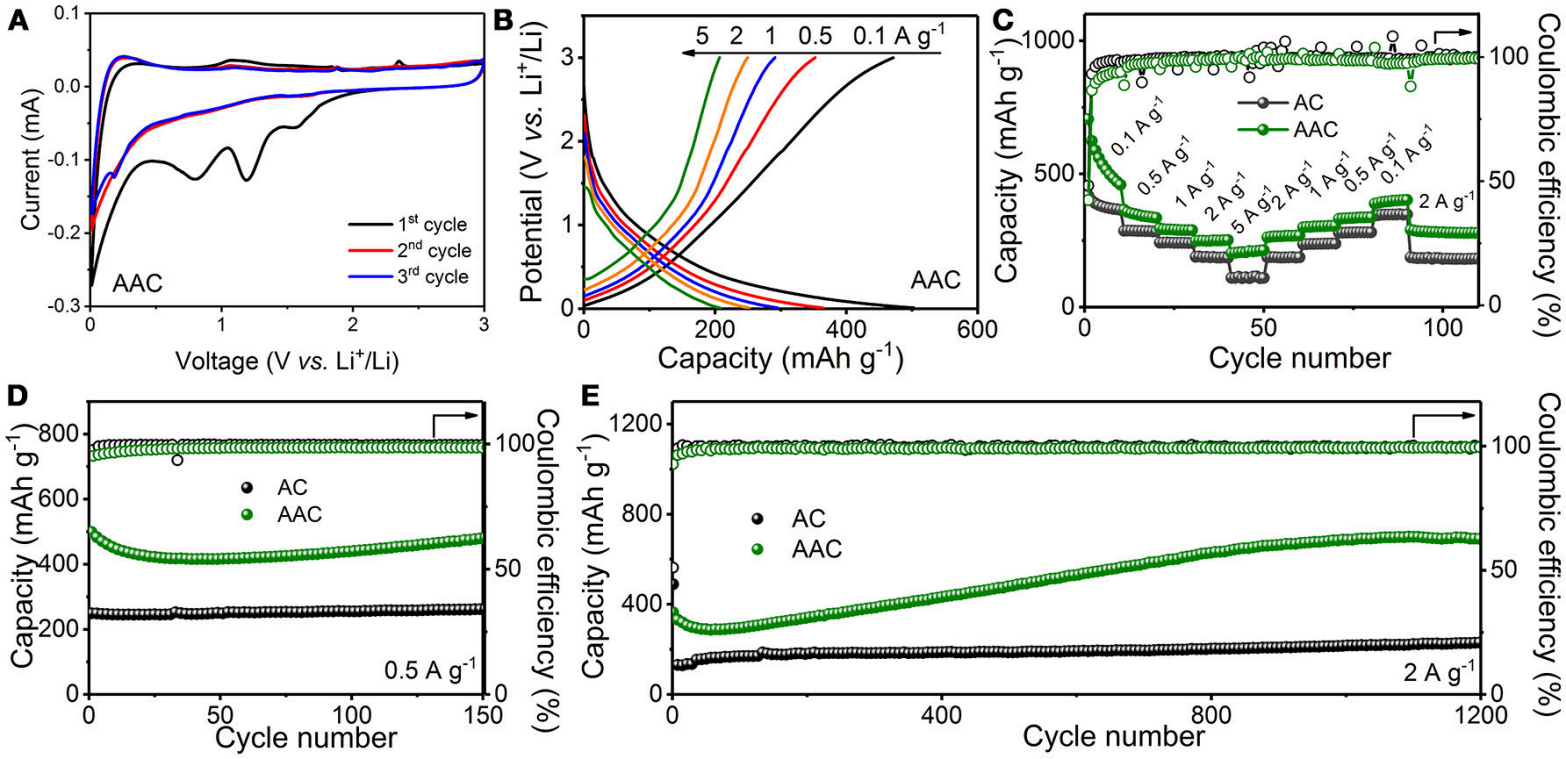
Electrochemical performances of AAC and AC as anode materials for LIBs: **(A)** cyclic voltammetry (CV) curves at 0.1 mV s^−1^ of AAC; **(B)** charge/discharge curves of AAC at different current densities; **(C)** rate performances; **(D,E)** long-term cyclic performances at 0.5 A g^−1^ **(D)** and 2 A g^−1^ **(E)**.

Figure 4 illustrates the electrochemical performances of AAC and AC as anode materials for LIBs. Electrochemical behaviors of AAC and AC during the initial three cycles are examined through cyclic voltammetry (CV) test at a scan rate of 0.1 mV s^−1^ within voltage range between 0.01 and 3.0 V, as presented at Figure 4A and Figure S3A, respectively. Like AC sample, three identifiable cathodic peaks for AAC, which center at range of 1.8–0.4 V in the first CV curves and vanish in the following cycles are ascribed to the formation of irreversible solid electrolyte interface (SEI) and unanticipated side reactions due to the consumption of oxygenic functional groups (Lv et al., 2015; Li et al., 2017b; Huang et al., 2018) Meanwhile, a reversible cathodic peak appears at potential window of 0.5–0.01 V, in concert with the anodic peak at 0.2 V, is originated from the reversible lithiation/delithiation process. It is observed that the 2nd and 3rd cycle of CV curves are scarcely discriminated, suggesting that SEI films formed at the first cycles securely protect the structural integrity of AAC an AC, and enduring the rapid ion insert/extraction. Correspondently, the first galvanostatic charge/discharge portrait at 0.1 A g^−1^ for AAC (Figure S2A) shows charge and discharge plateaus at around 1.6 and 1.2 V, respectively, which are generated from unavoidable SEI fabrication and electrolyte decomposition, leading to an irreversible capacity loss. Therefore, the charge and discharge capacities of AAC for the first cycle are 777.8 mAh g^−1^ and 1803.8 mAh g^−1^, with initial coulombic efficiency (ICE) about 43.12%, which is exceeding the AC anode.

Evaluation of rate performance for AAC anode is conducted by gradient current densities, as depicted in Figures 4B,C. AAC anode achieves the charge capacities of 459.7, 335.4, 288.7, 251.7, and 213.5 mAh g^−1^ at rate of 0.1, 0.5, 1, 2, and 5 A g^−1^, respectively, whereas AC delivers an overall inferior performance with unsatisfactory capacity decline at high current density of 5 A g^−1^ (Figure S3B). When the current density decreases from 5 to 0.1 A g^−1^ by stages, the charge capacity of AAC are restored to nearly 100% compared with the pristine state, reflecting the outstanding rate tolerance and stable structural maintenance of AAC. In galvanostatic charge/discharge test (Figures 4D,E), the charge capacity of AAC attractively reaches 479.2 mAh g^−1^ after 150 cycles at a current density of 0.5 A g^−1^ with 96% retention. Furthermore, it can achieve reversible capacity of 691.7 mAh g^−1^ after 1200 cycles at a high rate of 2 A g^−1^ with an ascending tendency in the first few hundred cycles, which is stemmed from gradual electrochemical activation of AAC and is similar to most carbonaceous anode materials (Qie et al., 2012; Lv et al., 2015; Xiong et al., 2018; Yang et al., 2018; Zhang et al., 2018b). On the contrary, ordinary AC merely delivers 261.6 mAh g^−1^ at 0.5 A g^−1^ and 229.1 mAh g^−1^ at 2 A g^−1^ under the same cycling condition. Conspicuous improvements in electrochemical performance for AAC anode can be explained by synergistic effort of highly porous structure and expanded interlayer of d_002_ plane due to KOH activation. Specifically, the interface between electrode and electrolyte is modified by multiple pores for wide wettability, facilitating a much more efficient accesses for ions transportation through minimizing inherent impedance and shorten the diffusion paths. Besides, the larger interlayer distance favors the reversibility of ion intercalation and extraction. Furthermore, taking the advantages of this unique structure, additional active sites for Li-ions adsorption are provided, and integral structure to mitigate volume variation are guaranteed, making AAC into a favorable anode material for LIBs (Qin et al., 2014; Xiong et al., 2016, 2017).

**Figure 5:**
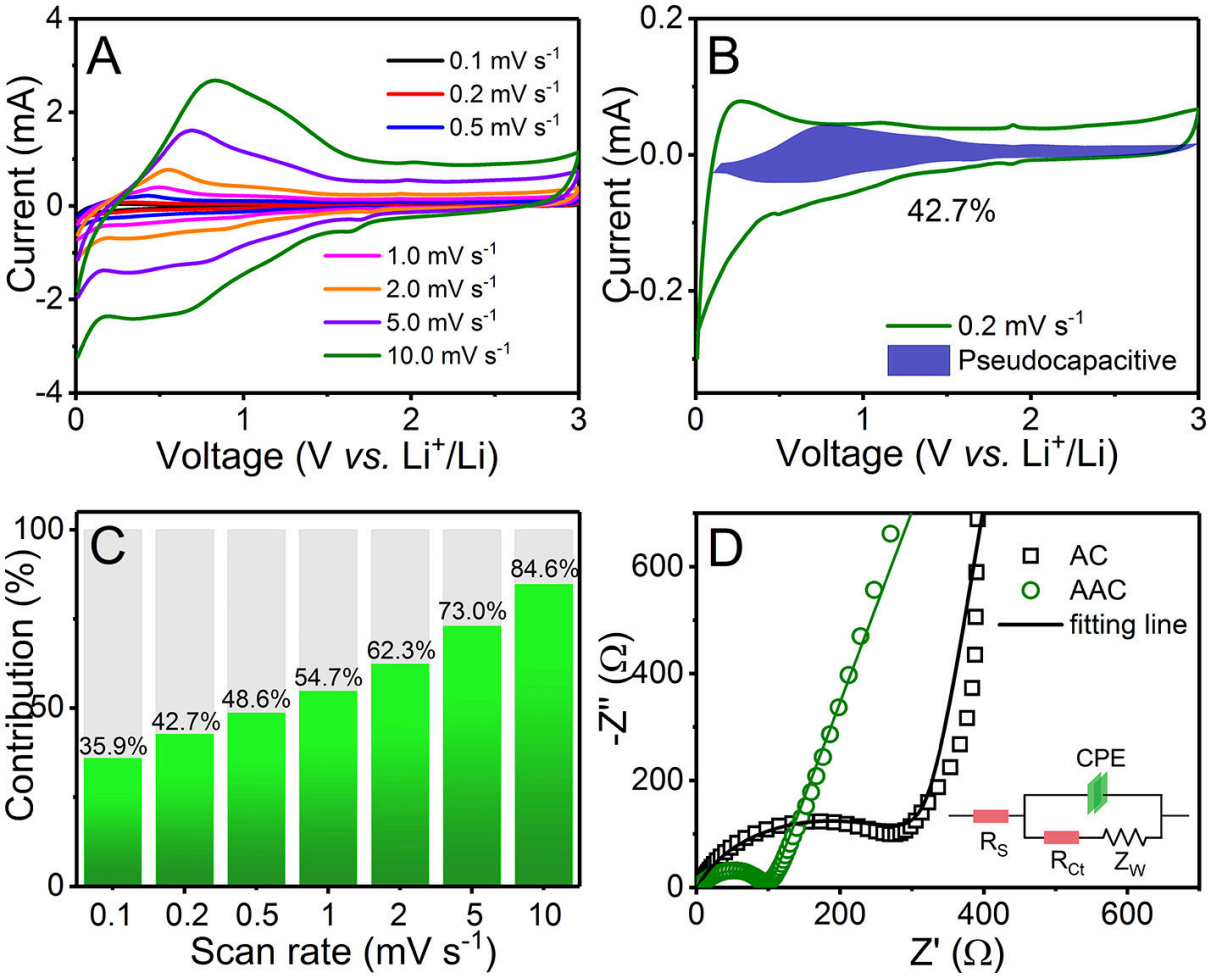
**(A)** CV curves measured between 0.01 and 3.0 V at different sweep rate from 0.1 to 10.0 mV s^−1^; **(B)** CV curve with a blue closed area representing pseudocapacitance at a sweep rate of 0.2 mV s^−1^; **(C)** bar chart of pseudocapacitive contribution at corresponding sweep rate; **(D)** electrochemical impedance spectra in form of Nyquist plots of AAC and AC before cycling with equivalent circuit diagram as the inset.

For further evidences, CV curves acquired at different scan rate varied from 0.1 to 10.0 mV s^−1^ with similar feature are shown in Figure 5A. Capacitive behavior of AAC is estimated *via* the following equation:

(1) $$\begin{array}{l} i\left(\text{V}\right)={\text{k}}_{1}\nu +{\text{k}}_{2}\nu^{1/2} \end{array}$$

or:

(2) $$\begin{array}{l} i\left(\text{V}\right)/\nu^{1/2}={\text{k}}_{1}\nu^{1/2}+{\text{k}}_{2} \end{array}$$

where *i* is the responsive current at various scan rates (*v*) at randomly chosen potential (V), and k (both k_1_ and k_2_) are constants. k_1_ can be attained by calculating the slope of the liner relationship between *i(V)/*ν^1/2^ and ν^1/2^ at every fixed potential, evaluating the proportion of charge storage from capacitance and calculating capacity contributions from pseudocapacitance and diffusion (Chen et al., 2017; Wang et al., 2018; Zhang et al., 2018a). As scan rate increases, capacitive distribution increases in the same time and achieves 42.7% at a scan rate of 0.2 mV s^−1^ (Figures 5B,C). When tested at a scan rate of 10 mV s^−1^, the capacitive distribution can reach as high as 84.6%. The Li^+^ ions are easy to make absorption or reactions in macro-micropores, resulting in the pseudocapacitive behavior at edges or on the surface of amorphous carbon. The higher ratio of pseudocapacitance allows charges to transfer in an effective way. With rational design and modification of AAC anode, excellent rate capability can be accomplished by reallocating the proportion of capacitive contribution to a more dominant occupancy. In order to confirm the smaller resistance of AAC, the electrochemical impedance spectroscopies (EIS) for AAC and AC anodes are carried out in Figure 5D. Obviously, the charge-transfer resistance (R_ct_) of AAC located in high frequency is much smaller than AC, reducing from 319.5 to 89.07 Ω, which results in the considerably improved electronic conductivity and reaction kinetics (Wu et al., 2016b, 2017). In accordance with capacity distribution, the effective rate response of AAC can be ascribed to the excellent reaction dynamics (Wang et al., 2018).

**Figure 6:**
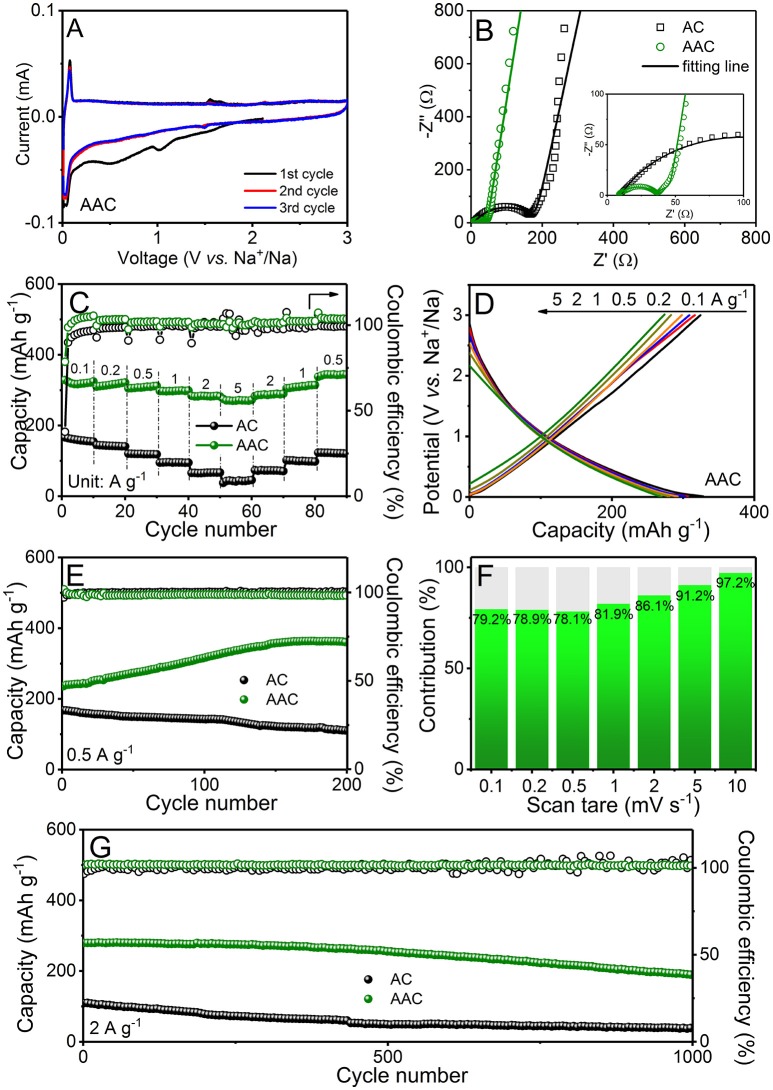
Electrochemical measurements of AAC and AC as anode materials for SIBs: **(A)** cyclic voltammetry (CV) curves at 0.1 mV s^−1^ of AAC; **(B)** electrochemical impedance spectra in form of Nyquist plots of AAC and AC before cycling with detailed view in high frequency area as the inset; **(C)** rate performances of AAC and AC and **(D)** charge/discharge curves of AAC at different current densities; **(E)** long-term cyclic performances at 0.5 A g^−1^; **(F)** bar chart of pseudocapacitive contribution at various sweep rate; **(G)** long-term cyclic performances at 2 A g^−1^.

The increasing price for Li-containing cathodes incents researchers to seek for alternative energy storage system, among which SIB is suitable and promising. Therefore, with the instruction of the employment in LIBs, the AAC can be also applied for SIBs (Figure 6). Figure 6A and Figure S3C present the CV curves of AAC and AC performed at a sweep rate of 0.1 mV s^−1^ within 0.01-3.0 V, respectively. Analyzing the CV curve of AAC anode (Figure 6A), it can observe a broad hump with two protuberant peaks at 1.0 and 0.4 V, which are disappeared in next two cycles. These irreversible peaks should be assigned to the formation of protective SEI film and undesirable reactions of oxygen-containing functional groups (Zhu et al., 2018), and this analogous phenomenon is also demonstrated in CV profiles of AC anode (Figure S3D) While a redox peak at 0.03 and 0.06 V is observed, referring to Na^+^-ion insertion/extraction. The overlapped CV profiles after the first cycle indicate a fully covered SEI film on AAC, suggesting the highly reversible redox reaction and stable structure. As shown in Figure S2B, AAC anode can exhibit an initial charge capacity of 328.7 mAh g^−1^ with the coulombic efficiency about 70%, while it maintains almost 100% columbic efficiency in the succeeding charge/discharge cycles. It is worth noticing that the initial coulombic efficiency of AAC is surprisingly higher than AC (Figure S2B), which can be reasonably elucidated to the activation of AAC. Based on adsorption-insertion sodium storage mechanism of amorphous carbon (Hou et al., 2017a; Li et al., 2017c), increasing amount of active sites (pores and defects) can attract more Na^+^ ion and the enlarged interlayer spacing is found suitable for Na^+^ intercalation/extraction between interlayers.

The comparison of AAC and AC anodes is measured sequentially at different current rates from 0.1 to 5 A g^−1^ (Figures 6C,D). The reversible capacities of AAC anode at every rate are much higher than AC anode, reaching to 324.8, 321.6, 312.1, 298.9, 282.3, and 272.4 mAh g^−1^ at 0.1, 0.2, 0.5, 1.0, 2.0, and 5.0 A g^−1^, respectively. Surprisingly, it can recover to 343.5 mAh g^−1^ when current density rolls back to 0.5 A g^−1^, indicating the superior rate performance. Besides, the long-term galvanostatic charge/discharge property of AAC and AC anodes are presented in Figures 6E,G. AAC anode obtains a charge capacity of 361.7 mAh g^−1^ at 0.5 A g^−1^ after 200 cycles and maintains 189.1 mAh g^−1^ at high rate of 2 A g^−1^ beyond 1000 cycles, surpassing the AC anode, which possesses awfully low charge capacity of only 113.1 mAh g^−1^ and 41.1 mAh g^−1^ at 0.5 A g^−1^ and 2 A g^−1^, respectively, with continuous degradation. The above enhanced cycling performance is originated from the three-dimension hierarchical porous structure equipped with well-fabricated SEI film as well as intrinsic shortened transport routes and facile intercalation for Na^+^ (Ou et al., 2017).

EIS measurements (Figure 6B) validate the excellent cyclic and rate capability of AAC by simulating the equivalent circuits. Figure 6B displays that AAC exhibits satisfactory low impedance R_ct_ of about 26.2 Ω, which is much smaller than that of AC (160.3 Ω), proving the low charge transfer resistance and fluent reaction process in AAC. Furthermore, the calculation of pseudocapacitive contributions at various sweep rates are also carried out in Figure 6F and Figure S4. Capacitive behavior of AAC is accountable for 78.9% of total capacity at sweep rate of 0.2 mV s^−1^, signifying that the critical capacitive behavior induced by larger specific surface area ensures the outstanding cyclic and rate performance of AAC. Compared with other published carbon anodes (Tables S1 and S2), it confirms that AAC exhibits attracting electrochemical performance for both lithium and sodium storage.

## Conclusion

In general, the activated amorphous carbon (AAC) with porous structure derived from renewable camellia pollen grains is synthesized by a facile approach of homogeneous KOH activation followed by carbonization. AAC exhibits the enhancement in specific surface area (660.04 m^2^ g^−1^), the enrichment in micro/mesopores (0.29 cm^3^ g^−1^ for accumulated pore volume) and the enlargement in interlayer distance (from 0.352 to 0.409 nm) as well. Furthermore, the modified microstructure can facilitate the infiltration for electrolyte into the interior of AAC electrode. On the other hand, it accesses the efficient transportation of Li^+^/Na^+^ ions by lowering the charge transfer resistance and shorten the diffusion paths. It is noted that the intercalation and adsorption of Li^+^/Na^+^ ion are coincidentally promoted, leading to the outstanding electrochemical performances. As a result, AAC anode surprisingly achieves a reversible charge capacity of 691.7 mAh g^−1^ after 1200 cycles at 2.0 A g^−1^ and 213.5 mAh g^−1^ at ultrahigh rate of 5.0 A g^−1^ for LIBs. Moreover, AAC delivers reversible capacities of 324.8, 321.6, 312.1, 298.9, 282.3, and 272.4 mAh g^−1^ at 0.1, 0.2, 0.5, 1.0, 2.0, and 5.0 A g^−1^ in gradient rate tests for SIBs. It has been discussed in plenty of articles that, concentration of activating reagent (KOH in this article) and activation duration are mutually responsible for the eventualities, which means that with rational adjustments can we delicately tailor the microstructure of pollen-grain-derived amorphous carbon. Alone with the particularity of pollen grains as a renewable and cost-effective biomass, the activated amorphous carbon with such intriguing structure is likely to become the future promising anode materials for lithium and sodium ion batteries.

## Author contributions

KX and JX conducted the experiments, CY is the supervisor of this research work. YL and XO helped writing. KX, YL, and XO performed the characterization and data analysis. All authors involved the analysis of experimental data and manuscript preparation.

### Conflict of interest statement

KX, YL, WS, and GZ were employed by company Electric Power Research Institute of Guangdong Power Grid Co., Ltd. The remaining authors declare that the research was conducted in the absence of any commercial or financial relationships that could be construed as a potential conflict of interest.

## References

[B1] Chen L.-F. Ma S.-X. Lu S. Feng Y. Zhang J. Xin S. (2016). Biotemplated synthesis of three-dimensional porous MnO/C-N nanocomposites from renewable rapeseed pollen: an anode material for lithium-ion batteries. Nano Res. 10, 1–11. 10.1007/s12274-016-1283-7

[B2] Chen Z. Wu R. Liu M. Wang H. Xu H. Guo Y. (2017). General synthesis of dual carbon-confined metal sulfides quantum dots toward high-performance anodes for sodium-ion batteries. Adv. Funct. Mater. 27:1702046 10.1002/adfm.201702046

[B3] Gao Z. Zhang Y. Song N. Li X. (2016). Biomass-derived renewable carbon materials for electrochemical energy storage. Mater. Res. Lett. 5, 69–88. 10.1080/21663831.2016.1250834

[B4] Gu X. Wang Y. Lai C. Qiu J. Li S. Hou Y. (2014). Microporous bamboo biochar for lithium-sulfur batteries. Nano Res. 8, 129–139. 10.1007/s12274-014-0601-1

[B5] Hannan M. A. Lipu M. S. H. Hussain A. Mohamed A. (2017). A review of lithium-ion battery state of charge estimation and management system in electric vehicle applications: challenges and recommendations. Renew. Sust. Energ. Rev. 78, 834–854. 10.1016/j.rser.2017.05.001

[B6] Hou H. Banks C. E. Jing M. Zhang Y. Ji X. (2015). Carbon quantum dots and their derivative 3D porous carbon frameworks for sodium-ion batteries with ultralong cycle life. Adv. Mater. 27, 7861–7866. 10.1002/adma.201503816 26506218

[B7] Hou H. Qiu X. Wei W. Zhang Y. Ji X. (2017a). Carbon anode materials for advanced sodium-ion batteries. Adv. Energy. Mater. 7:1602898 10.1002/aenm.201602898

[B8] Hou H. Shao L. Zhang Y. Zou G. Chen J. Ji X. (2017b). Large-Area carbon nanosheets doped with phosphorus: a high-performance anode material for sodium-ion batteries. Adv. Sci. 4:1600243. 10.1002/advs.201600243 28105399 PMC5238737

[B9] Huang S. Li Z. Wang B. Zhang J. Peng Z. Qi R. (2018). N-Doping and defective nanographitic domain coupled hard carbon nanoshells for high performance lithium/sodium storage. Adv. Funct. Mater. 28:1706294 10.1002/adfm.201706294

[B10] Irisarri E. Ponrouch A. Palacin M. R. (2015). Review-hard carbon negative electrode materials for sodium-ion batteries. J. Electrochem. Soc. 162, A2476–A2482. 10.1149/2.0091514jes

[B11] Kim H. S. Cook J. B. Lin H. Ko J. S. Tolbert S. H. Ozolins V.. (2017). Oxygen vacancies enhance pseudocapacitive charge storage properties of MoO_3−x_. Nat. Mater. 16, 454–460. 10.1038/nmat4810 27918566

[B12] Li R. Huang J. Xu Z. Qi H. Cao L. Liu Y. (2017a). Controlling the thickness of disordered turbostratic nanodomains in hard carbon with enhanced sodium storage performance. Energy Tech. 6, 1080–1087. 10.1002/ente.201700674

[B13] Li Y. Hu Y.-S. Titirici M.-M. Chen L. Huang X. (2016). Hard carbon microtubes made from renewable cotton as high-performance anode material for sodium-ion batteries. Adv. Energy Mater. 6:1600659 10.1002/aenm.201600659

[B14] Li Y. Xu S. Wu X. Yu J. Wang Y. Hu Y.-S. (2015). Amorphous monodispersed hard carbon micro-spherules derived from biomass as a high performance negative electrode material for sodium-ion batteries. J. Mater. Chem. A 3, 71–77. 10.1039/C4TA05451B

[B15] Li Y.-Q. Li J.-C. Lang X.-Y. Wen Z. Zheng W.-T. Jiang Q. (2017b). Lithium ion breathable electrodes with 3D hierarchical architecture for ultrastable and high-capacity lithium storage. Adv. Funct. Mater. 27:1700447 10.1002/adfm.201700447

[B16] Li Z. Bommier C. Chong Z. S. Jian Z. Surta T. W. Wang X. (2017c). Mechanism of Na-ion storage in hard carbon anodes revealed by heteroatom doping. Adv. Energy Mater. 7:1602894 10.1002/aenm.201602894

[B17] Li Z. Xu Z. Tan X. Wang H. Holt C. M. B. Stephenson T. (2013). Mesoporous nitrogen-rich carbons derived from protein for ultra-high capacity battery anodes and supercapacitors. Energy Environ. Sci. 6, 871–878. 10.1039/c2ee23599d

[B18] Lin D. Liu Y. Liang Z. Lee H. W. Sun J. Wang H.. (2016). Layered reduced graphene oxide with nanoscale interlayer gaps as a stable host for lithium metal anodes. Nat. Nanotechnol. 11, 626–632. 10.1038/nnano.2016.32 26999479

[B19] Luo W. Shen F. Bommier C. Zhu H. Ji X. Hu L. (2016). Na-ion battery anodes: materials and electrochemistry. Acc. Chem. Res. 49, 231–240. 10.1021/acs.accounts.5b00482 26783764

[B20] Lv W. Wen F. Xiang J. Zhao J. Li L. Wang L. (2015). Peanut shell derived hard carbon as ultralong cycling anodes for lithium and sodium batteries. Electrochim. Acta 176, 533–541. 10.1016/j.electacta.2015.07.059

[B21] Ou X. Xiong X. Zheng F. Yang C. Lin Z. Hu R. (2016). *In situ* X-ray diffraction characterization of NbS_2_ nanosheets as the anode material for sodium ion batteries. J. Power Sources 325, 410–416. 10.1016/j.jpowsour.2016.06.055

[B22] Ou X. Yang C. Xiong X. Zheng F. Pan Q. Jin C. (2017). A new rGO-overcoated Sb_2_Se_3_ nanorods anode for Na^+^ battery: *in situ* X-ray diffraction study on a live sodiation/desodiation process. Adv. Funct. Mater. 27:1606242 10.1002/adfm.201606242

[B23] Qie L. Chen W. M. Wang Z. H. Shao Q. G. Li X. Yuan L. X.. (2012). Nitrogen-doped porous carbon nanofiber webs as anodes for lithium ion batteries with a superhigh capacity and rate capability. Adv. Mater. 24, 2047–2050. 10.1002/adma.201104634 22422374

[B24] Qin J. He C. Zhao N. Wang Z. Shi C. Liu E.. (2014). Graphene networks anchored with Sn@graphene as lithium ion battery anode. Acs Nano 8, 1728–1738. 10.1021/nn406105n 24400945

[B25] Romanos J. Beckner M. Rash T. Firlej L. Kuchta B. Yu P.. (2012). Nanospace engineering of KOH activated carbon. Nanotechnology 23:015401. 10.1088/0957-4484/23/1/015401 22156024

[B26] Shen C. Long H. Wang G. Lu W. Shao L. Xie K. (2018). Na_3_V_2_(PO_4_)_2_F_3_@C dispersed within carbon nanotube frameworks as a high tap density cathode for high-performance sodium-ion batteries. J. Mater. Chem. A 6, 6007–6014. 10.1039/C8TA00990B

[B27] Su H. Zhang H. Liu F. Chun F. Zhang B. Chu X. (2017). High power supercapacitors based on hierarchically porous sheet-like nanocarbons with ionic liquid electrolytes. Chem. Eng. J. 322, 73–81. 10.1016/j.cej.2017.04.012

[B28] Tang J. Etacheri V. Pol V. G. (2016). From allergens to battery anodes: nature-inspired, pollen derived carbon architectures for room- and elevated-temperature Li-ion storage. Sci. Rep. 6:20290. 10.1038/srep20290 26846311 PMC4742870

[B29] Wang J. Kaskel S. (2012). KOH activation of carbon-based materials for energy storage. J. Mater. Chem. 22, 23710–23725. 10.1039/c2jm34066f

[B30] Wang X. Li G. Hassan F. M. Li J. Fan X. Batmaz R. (2015). Sulfur covalently bonded graphene with large capacity and high rate for high-performance sodium-ion batteries anodes. Nano Energy 15, 746–754. 10.1016/j.nanoen.2015.05.038

[B31] Wang Y. Kong D. Huang S. Shi Y. Ding M. Von Lim Y. (2018). 3D carbon foam-supported WS_2_ nanosheets for cable-shaped flexible sodium ion batteries. J. Mater. Chem. A 6, 10813–10824. 10.1039/C8TA02773K

[B32] Wen Y. He K. Zhu Y. Han F. Xu Y. Matsuda I.. (2014). Expanded graphite as superior anode for sodium-ion batteries. Nat. Commun. 5:4033. 10.1038/ncomms5033 24893716

[B33] Wu L. Buchholz D. Vaalma C. Giffin G. A. Passerini S. (2016a). Apple biowaste-derived hard carbon as powerful anode material for Na-ion batteries. Chemelectrochem 3, 292–298. 10.1002/celc.201500437

[B34] Wu X. Li Y. Xiang Y. Liu Z. He Z. Wu X. (2016b). The electrochemical performance of aqueous rechargeable battery of Zn/Na_0.44_MnO_2_ based on hybrid electrolyte. J. Power Sources 336, 35–39. 10.1016/j.jpowsour.2016.10.053

[B35] Wu X. Xiang Y. Peng Q. Wu X. Li Y. Tang F. (2017). Green-low-cost rechargeable aqueous zinc-ion batteries using hollow porous spinel ZnMn_2_O_4_ as the cathode material. J. Mater. Chem. A 5, 17990–17997. 10.1039/C7TA00100B

[B36] Xiong J. Pan Q. Zheng F. Xiong X. Yang C. Hu D.. (2018). N/S co-doped carbon derived from cotton as high performance anode materials for lithium ion batteries. Front. Chem. 6:78. 10.3389/fchem.2018.00078 29755966 PMC5932144

[B37] Xiong X. Wang G. Lin Y. Wang Y. Ou X. Zheng F.. (2016). Enhancing sodium ion battery performance by strongly binding nanostructured Sb_2_S_3_ on sulfur-doped graphene sheets. ACS Nano 10, 10953–10959. 10.1021/acsnano.6b05653 27930883

[B38] Xiong X. Yang C. Wang G. Lin Y. Ou X. Wang J.-H. (2017). SnS nanoparticles electrostatically anchored on three-dimensional N-doped graphene as an active and durable anode for sodium-ion batteries. Energy Environ. Sci. 10, 1757–1763. 10.1039/C7EE01628J

[B39] Yang C. Ou X. Xiong X. Zheng F. Hu R. Chen Y. (2017). V_5_S_8_-graphite hybrid nanosheets as a high rate-capacity and stable anode material for sodium-ion batteries. Energy Environ. Sci. 10, 107–113. 10.1039/C6EE03173K

[B40] Yang C. Xiong J. Ou X. Wu C.-F. Xiong X. Wang J.-H. (2018). A renewable natural cotton derived and nitrogen/sulfur co-doped carbon as a high-performance sodium ion battery anode. Mater. Today Energy 8, 37–44. 10.1016/j.mtener.2018.02.001

[B41] Yu W. Wang H. Liu S. Mao N. Liu X. Shi J. (2016). N, O-codoped hierarchical porous carbons derived from algae for high-capacity supercapacitors and battery anodes. Mater. Chem. A 4, 5973–5983. 10.1039/C6TA01821A

[B42] Zhang N. Wang Y. Jia M. Liu Y. Xu J. Jiao L.. (2018a). Ultrasmall Sn nanoparticles embedded in spherical hollow carbon for enhanced lithium storage properties. Chem. Commun. 54, 1205–1208. 10.1039/C7CC09095A 29335702

[B43] Zhang Q. Chen H. Luo L. Zhao B. Luo H. Han X. (2018b). Harnessing the concurrent reaction dynamics in active Si and Ge to achieve high performance lithium-ion batteries. Energy Environ. Sci. 11, 669–681. 10.1039/C8EE00239H

[B44] Zhang Q. Han K. Li S. Li M. Li J. Ren K. (2018c). Synthesis of garlic skin-derived 3D hierarchical porous carbon for high-performance supercapacitors. Nanoscale 10, 2427–2437. 10.1039/C7NR07158B 29335695

[B45] Zhang R. Li N. W. Cheng X. B. Yin Y. X. Zhang Q. Guo Y. G. (2017). Advanced micro/nanostructures for lithium metal anodes. Adv. Sci. 4:1600445. 10.1002/advs.201600445 28331792 PMC5357990

[B46] Zhang Y. Foster C. W. Banks C. E. Shao L. Hou H. Zou G.. (2016b). Graphene-Rich wrapped petal-like rutile TiO_2_ tuned by carbon dots for high-performance sodium storage. Adv. Mater. 28, 9391–9399. 10.1002/adma.201601621 27573868

[B47] Zhang Y.-C. You Y. Xin S. Yin Y.-X. Zhang J. Wang P. (2016a). Rice husk-derived hierarchical silicon/nitrogen-doped carbon/carbon nanotube spheres as low-cost and high-capacity anodes for lithium-ion batteries. Nano Energy 25, 120–127. 10.1016/j.nanoen.2016.04.043

[B48] Zheng F. Yang C. Xiong X. Xiong J. Hu R. Chen Y.. (2015). Nanoscale surface modification of lithium-rich layered-oxide composite cathodes for suppressing voltage fade. Angew. Chem. Int. Ed. Engl. 54, 13058–13062. 10.1002/anie.201506408 26335589

[B49] Zhu Z. Liang F. Zhou Z. Zeng X. Wang D. Dong P. (2018). Expanded biomass-derived hard carbon with ultra-stable performance in sodium-ion batteries. J. Mater. Chem. A 6, 1513–1522. 10.1039/C7TA07951F

[B50] Zou G. Wang C. Hou H. Wang C. Qiu X. Ji X. (2017). Controllable interlayer spacing of sulfur-doped graphitic carbon nanosheets for fast sodium-ion batteries. Small 13:1700762. 10.1002/smll.201700762 28650567

