# Supplementary material for: Activated Amorphous Carbon With High-Porosity Derived From Camellia Pollen Grains as Anode Materials for Lithium/Sodium Ion Batteries
Source: Front Chem. 2018 Sep 4;6:366. doi: 10.3389/fchem.2018.00366 (PMC6131591; doi:10.3389/fchem.2018.00366)
Supplement: Supplementary file 1 [file Data_Sheet_1.doc]

# Supplementary Material

Figure S1. SEM images of (A-B) camellia pollen grains and (C-D) pristine amorphous carbon.

Figure S2. initial charge/discharge profiles of AAC and AC in (A) LIB and (B) SIB.

Figure S3. (A) CV curves at 0.1 mV s^-1^ and (B) charge/discharge profiles at different current densities of AC in LIB; (C) CV curves at 0.1 mV s^-1^ and (D) charge/discharge profiles at different current densities of AC in SIB.

Figure S4. CV curves of AAC at a series of sweep rate from 0.1 to 10 mV s^-1^; CV curves of AAC with enclosed blue area for pseudocapacitive contribution at 0.2 mV s^-1^.

**Table S1.** A comparison with literatures of the reversible capacities for carbonaceous materials, tested in half-cell configuration vs. Li.

| Materials | Sources | Rate performance (mAh g^-1^) | Cycling Stability (mAh g^-1^) | References |
| --- | --- | --- | --- | --- |
| carbon fiber | rice husk | 137 at 3.75 A g^-1^ | 403 at 0.075 A g^-1^ after100 cycles | S1 |
| activated carbon | cattail pollens | ~200 at 0.372 A g^-1^ | 382 at 0.037 A g^-1^ after 50 cycles | S2 |
| porous carbon spheres | sucrose | 270 at 0.372 A g^-1^ | 365 at 0.037 A g^-1^ after 100 cycles;  250 at 0. 372 A g^-1^ after 100 cycles | S3 |
| amorphou spheroidal carbon | coffee oil | ~160 at 0.5 A g^-1^ | 274 at 0.1 A g^-1^ after 250 cycles | S4 |
| carbon | waste-loofah | 204 at 1.0 A g^-1^ | 187 at at 1 A g^-1^ after 500 cycles;  98 at at 3 A g^-1^ after 500 cycles | S5 |
| activated amorphous carbon | camellia pollen grains | 213.5 at 5.0 A g^-1^ | 479.2 at 0.5 A g^-1^ after 150 cycles;  691.7 at 2 A g^-1^ after 1200 cycles | Our work |

**Table S2.** A comparison with literatures of the reversible capacities for carbonaceous materials, tested in half-cell configuration vs. Na.

| Materials | Sources | Rate performance (mAh g^-1^) | Cycling Stability (mAh g^-1^) | References |
| --- | --- | --- | --- | --- |
| hard carbon | cherry petals | 41.7 at 5.0 A g^-1^ | 298.1 at 0.02 A g^-1^ after 100 cycles | S6 |
| hard carbon | biomass humic acid | 62 at 10.0 A g^-1^ | 208 at 0.1 A g^-1^ after 250 cycles | S7 |
| hard carbon | kelp | 96 at 1 A g^-1^ | 205 at 0.2 A g^-1^ after 300 cycles | S8 |
| carbon | pistachio shell | ~80 at 0.2 A g^-1^ | 130 at 0.04 A g^-1^ after 50 cycles | S9 |
| hard carbon microtubes | cotton | ~90 at 0.6 A g^-1^ | 305 at 0.03 A g^-1^ after 100 cycles | S10 |
| activated amorphous carbon | camellia pollen grains | 272.4 at 5.0 A g^-1^ | 361.7 at 0.5 A g^-1^ after 200 cycles;  189.1 at 2 A g^-1^  after 1000 cycles | Our work |

**References**

S1. Wang, L., Schnepp, Z., and Titirici, M.M. (2013). Rice husk-derived carbon anodes for lithium ion batteries. *Journal of Materials Chemistry A* 1**,** 5269. doi: 10.1039/c3ta10650k

S2. Tang, J., Etacheri, V., and Pol, V.G. (2016). From allergens to battery anodes: nature-inspired, pollen derived carbon architectures for room- and elevated-temperature Li-ion storage. *Sci Rep* 6**,** 20290. doi: 10.1038/srep20290

S3. Etacheri, V., Wang, C., O'connell, M.J., Chan, C.K., and Pol, V.G. (2015). Porous carbon sphere anodes for enhanced lithium-ion storage. *Journal of Materials Chemistry A* 3**,** 9861-9868. doi: 10.1039/c5ta01360g

S4. Kim, K., Adams, R.A., Kim, P.J., Arora, A., Martinez, E., Youngblood, J.P., and Pol, V.G. (2018). Li-ion storage in an amorphous, solid, spheroidal carbon anode produced by dry-autoclaving of coffee oil. *Carbon* 133**,** 62-68. doi: 10.1016/j.carbon.2018.03.013

S5. Hou, H., Yu, C., Liu, X., Yao, Y., Liao, Q., Dai, Z., and Li, D. (2018). Waste-loofah-derived carbon micro/nanoparticles for lithium ion battery anode. *Surface Innovations* 6**,** 159-166. doi: 10.1680/jsuin.17.00068

S6. Zhu, Z., Liang, F., Zhou, Z., Zeng, X., Wang, D., Dong, P., Zhao, J., Sun, S., Zhang, Y., and Li, X. (2018). Expanded biomass-derived hard carbon with ultra-stable performance in sodium-ion batteries. *Journal of Materials Chemistry A* 6**,** 1513-1522. doi: 10.1039/c7ta07951f

S7. Zhu, Y., Chen, M., Li, Q., Yuan, C., and Wang, C. (2017). High-yield humic acid-based hard carbons as promising anode materials for sodium-ion batteries. *Carbon* 123**,** 727-734. doi: 10.1016/j.carbon.2017.08.030

S8. Wang, P., Zhu, X., Wang, Q., Xu, X., Zhou, X., and Bao, J. (2017). Kelp-derived hard carbons as advanced anode materials for sodium-ion batteries. *Journal of Materials Chemistry A* 5**,** 5761-5769. doi: 10.1039/c7ta00639j

S9. Kim, K., Lim, D.G., Han, C.W., Osswald, S., Ortalan, V., Youngblood, J.P., and Pol, V.G. (2017). Tailored carbon anodes derived from biomass for sodium-ion storage. *ACS Sustainable Chemistry & Engineering* 5**,** 8720-8728. doi: 10.1021/acssuschemeng.7b01497

S10. Li, Y., Hu, Y.-S., Titirici, M.-M., Chen, L., and Huang, X. (2016). Hard carbon microtubes made from renewable cotton as high-performance anode material for sodium-ion batteries. *Advanced Energy Materials* 6**,** 1600659. doi: 10.1002/aenm.201600659
